# Association between treatment decisions and survival outcomes in lung cancer patients under a multidisciplinary team model: the impact of MDT-recommended treatment plans on treatment completion rates, survival time, and treatment conversion

**DOI:** 10.3389/fonc.2026.1793539

**Published:** 2026-05-04

**Authors:** Qian Yi, Hailong Wei

**Affiliations:** Department of Respiratory and Critical Care Medicine, the People’s Hospital of Leshan, Leshan, Sichuan, China

**Keywords:** lung cancer, multidisciplinary team, survival analysis, treatment adherence, treatment decision

## Abstract

**Background:**

The complexity of lung cancer management requires coordinated care. This study aimed to evaluate the impact of Multidisciplinary Team (MDT)-recommended treatment plans on the treatment course and survival outcomes of lung cancer patients.

**Methods:**

This single-center, retrospective cohort study enrolled consecutive patients with primary lung cancer who were initially diagnosed and received first anti-cancer treatment between January 1, 2020, and January 30, 2023. Patients were categorized into MDT (n=115) or non-MDT (n=105) groups based on whether the initial treatment decision was made via a formal MDT discussion. Key measures included treatment completion rate, treatment conversion, overall survival (OS), progression-free survival (PFS), and safety profiles. Follow-up data were collected until July 31, 2025.

**Results:**

The MDT group demonstrated a significantly higher rate of completing the initial treatment as planned (86.09% vs. 68.57%, P = 0.002), a lower treatment conversion rate (19.13% vs. 39.05%, P = 0.001), and a longer time to conversion (8.41 vs. 5.16 months, P<0.001). Patients in the MDT group had significantly longer median OS (45.21 vs. 28.64 months, P<0.001) and PFS (19.80 vs. 13.53 months, P<0.001). Multivariate analyses confirmed MDT participation as an independent factor associated with improved OS (HR = 0.605, P = 0.009) and a higher likelihood of treatment completion (OR = 3.438, P = 0.002).

**Conclusion:**

The MDT model is associated with better treatment adherence, delayed treatment conversion, and significantly improved survival outcomes in lung cancer patients, and facilitates more precise initial treatment selection.

## 1 Introduction

Lung cancer persists as the leading cause of cancer-related mortality worldwide, presenting a formidable challenge to healthcare systems. Its management is inherently complex, characterized by a diverse spectrum of histopathological subtypes, molecular profiles, and disease stages, each necessitating a distinct therapeutic pathway (1, 2). The evolution of treatment modalities—encompassing surgery, radiotherapy, conventional chemotherapy, targeted therapy, and immunotherapy—has significantly expanded the therapeutic arsenal (3, 4). However, this expansion has concurrently intensified the intricacy of clinical decision-making, as the optimal initial treatment strategy is rarely dictated by a single factor but rather emerges from a nuanced synthesis of tumor biology, disease extent, patient performance status, and genomic data (5, 6).

The formulation of an effective treatment plan thus demands expertise spanning multiple, distinct medical disciplines. Traditionally, care pathways have been siloed, with patients navigating sequential consultations across different specialties. This fragmented approach risks generating conflicting recommendations, creating confusion for both patients and clinicians, and potentially leading to suboptimal or delayed treatment initiation (7). The critical need for a cohesive, patient-centric strategy in this multifaceted landscape has driven the adoption of integrated care models to bridge these disciplinary gaps (8).

In response, the Multidisciplinary Team (MDT) model has emerged as a cornerstone of contemporary oncology practice. This model orchestrates a structured, concurrent discussion among core specialists—including thoracic surgeons, medical and radiation oncologists, radiologists, and pathologists—to review all relevant diagnostic information for a specific patient (9, 10). The collaborative objective is to achieve consensus on a unified, evidence-based treatment recommendation that aligns with both clinical guidelines and individual patient circumstances. The theoretical benefits are substantial: MDT discussions aim to minimize bias, enhance diagnostic accuracy, improve staging precision, and promote the selection of the most appropriate initial therapy, particularly for cases with clinical ambiguity or where multiple valid options exist (11, 12).

While the conceptual value of MDTs is widely endorsed, and a growing body of observational evidence suggests a correlation between MDT review and improved survival outcomes in various cancers, including lung cancer, the mechanisms underlying this potential benefit require deeper exploration (13, 14). Much of the existing literature focuses predominantly on survival as the primary endpoint. Crucially, there is a relative paucity of detailed analysis regarding how MDT-formulated plans directly influence the process of care delivery (15). Key intermediate outcomes, such as the likelihood of patients completing their planned treatment regimen, the frequency and timing of unplanned treatment modifications (conversion), and the patterns of initial therapy selection, remain underexamined (16). Understanding these process metrics is essential, as they represent the direct, tangible manifestation of MDT decisions and likely serve as critical mediators between the collaborative decision-making event and the ultimate survival outcome.

Therefore, this study aims to systematically evaluate the association between MDT-driven treatment decisions and both the treatment journey and long-term prognosis in lung cancer patients. Specifically, we seek to investigate the impact of an MDT-recommended treatment plan on the treatment completion rate, survival time (overall and progression-free), and the incidence and timing of treatment conversion, thereby providing a more comprehensive assessment of the MDT model’s effectiveness in real-world clinical practice.

## 2 Materials and methods

### 2.1 Study subjects and screening criteria

This study is a single-center, retrospective cohort study. Through the hospital’s electronic medical record system, patients with primary lung cancer who were initially diagnosed and received their first anti-cancer treatment at our institution between January 1, 2020, and January 30, 2023, were consecutively screened.

Inclusion criteria were: ① Pathologically confirmed primary lung cancer (non-small cell lung cancer or small cell lung cancer); ② No prior anti-tumor treatment at the time of initial diagnosis; ③ Age ≥18 years; ④ Complete baseline clinical data, treatment records, and follow-up data available. Exclusion criteria included: ① Co-morbid severe mental illness or cognitive impairment; ② Co-morbid severe heart, liver, kidney dysfunction, or hematopoietic system diseases; ③ Severe missing clinical data that precludes evaluation of the treatment process and outcomes; ④ Concurrent active malignancies other than lung cancer; ⑤ Pregnant or breastfeeding women; ⑥ Loss to follow-up after the first treatment or overall survival follow-up less than three months as of the data analysis date (July 31, 2025).

**Figure 1:**
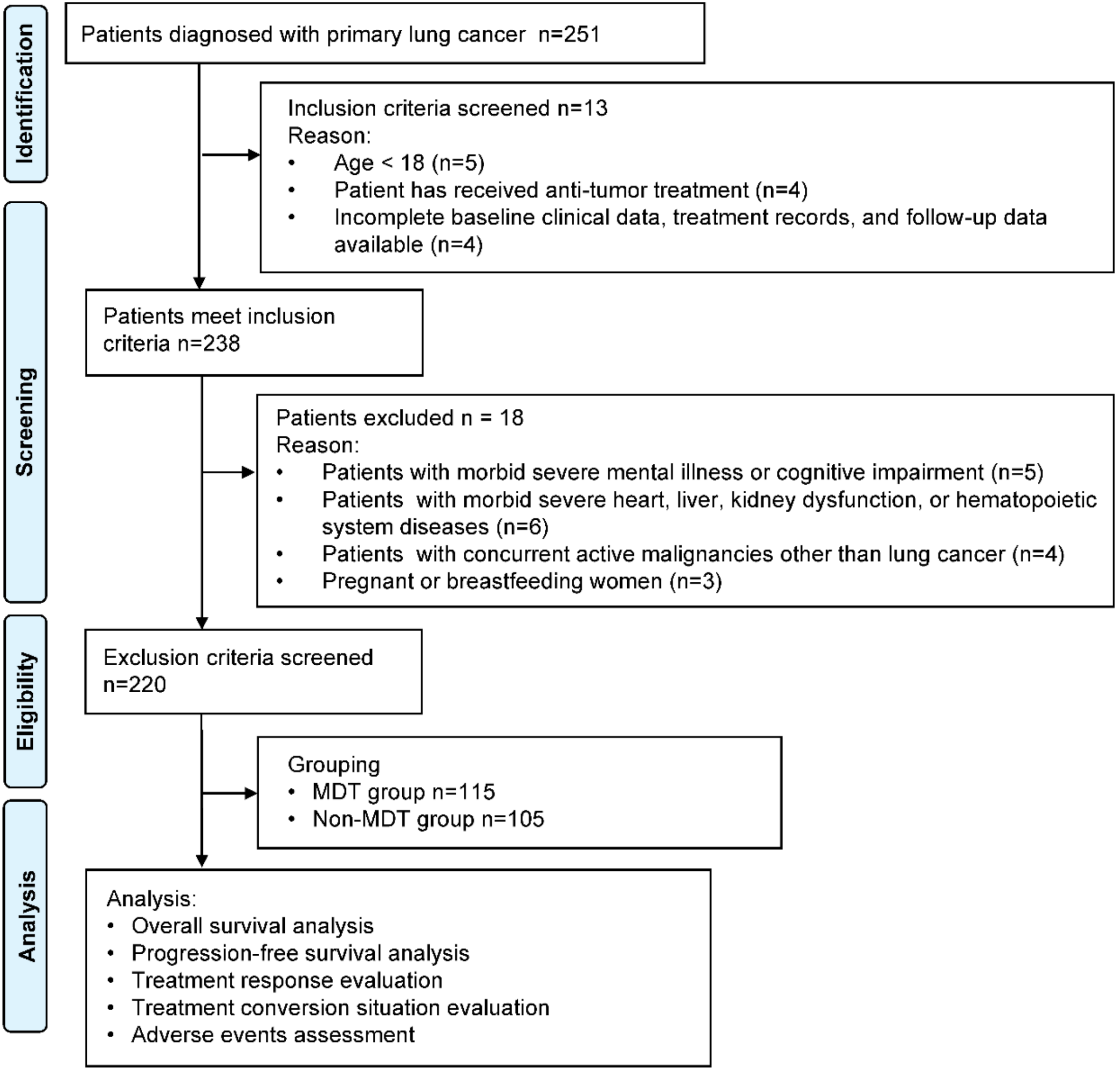
Flowchart of patient selection.

According to whether the initial treatment decision was made through a formally organized multidisciplinary team (MDT) discussion at the hospital, 220 eligible patients were divided into the MDT group (n=115) and the non-MDT group (n=105) (**Figure 1**). The assignment to MDT discussion was not randomized but reflected real-world clinical practice. Some patients were not discussed due to urgent presentations requiring immediate treatment, missed MDT meetings, or patient preference after being informed of the MDT option. For the MDT group, all clinical data of the patients were discussed during weekly regular MDT meetings before treatment. Core members of the MDT consistently included thoracic surgeons (n=3), medical oncologists (n=4), radiation oncologists (n=2), radiologists (n=2), and pathologists (n=2), and a nurse coordinator. The consensus treatment plan reached during the discussions was documented as the “MDT-recommended plan”. In contrast, for the non-MDT group, the initial treatment decisions were made independently by the primary specialist physician or through sequential consultations with multiple specialists without undergoing the aforementioned formal, concurrent MDT meeting.

### 2.2 Ethical statement

This study was conducted in accordance with the Helsinki Declaration and was approved by the Institutional Review Board (IRB) of our hospital. We performed a retrospective analysis of anonymized clinical data extracted from electronic medical records without imposing any additional interventions on the patients’ treatment processes. The IRB waived the requirement for informed consent.

### 2.3 Treatment decision-making process

#### 2.3.1 MDT group process

For patients in the MDT group, prior to initiating treatment, all relevant clinical data (imaging, pathology, performance status) were reviewed in a weekly scheduled MDT meeting. A consensus recommendation for the primary treatment plan (e.g., surgery with or without neoadjuvant or adjuvant therapy, definitive chemoradiation, systemic therapy alone) was documented in the electronic health record. The recommended treatment plan, along with potential alternative options and a suggested follow-up/transition plan upon disease progression, was communicated to the patient.

#### 2.3.2 Non-MDT group process

For patients in the Non-MDT group, the initial treatment plan was determined by the first consulting specialist or through sequential consultations without a structured, concurrent discussion among all relevant disciplines. The treatment plan was based on the individual specialist’s judgment and documented accordingly. Although institutional policy encourages MDT discussion for all newly diagnosed lung cancer cases, it was not strictly enforced during the early study period. Some patients received surgery or radical radiotherapy without prior MDT discussion due to urgent symptomatic presentation, rapid disease progression, or direct referral to surgical/radiation oncology services before the scheduled MDT meeting.

### 2.4 Research variables and data collection

Data were independently extracted by two trained researchers from the hospital’s electronic medical record system, MDT meeting records system, and the picture archiving and communication system (PACS). Discrepancies were resolved by a third senior researcher.

1. Baseline characteristics included age, gender, smoking history, pathological type, TNM stage (8th edition, assessed at initial diagnosis), performance status score (ECOG PS), and major comorbidities.
2. EGFR mutation analysis was performed using amplification-refractory mutation system polymerase chain reaction (ARMS-PCR) on a SLAN-96S Real-Time PCR System (Shanghai Hongshi Medical Technology Co., Ltd., China). ALK rearrangement was detected by Ventana anti-ALK (D5F3) immunohistochemistry on a BenchMark ULTRA automatic staining instrument (Roche Diagnostics, Basel, Switzerland). All patients underwent both tests as part of routine diagnostic workup.
3. Treatment completion rate: Defined as the proportion of patients who completed the initial treatment plan as scheduled (e.g., planned number of chemotherapy cycles, total radiation dose, surgical extent) without unplanned discontinuation due to disease progression, intolerable toxicity, patient refusal, or other non-medical reasons.
4. Treatment conversion: Recorded whether there was any change in the treatment plan during the first course of treatment due to any reason (e.g., poor efficacy, toxicity, patient preference), along with the time of conversion.
5. MDT adherence (MDT group only): Assessed the consistency between the actual treatment plan and the MDT-recommended plan (fully consistent/partially adjusted/completely inconsistent).
6. Survival time: Overall survival (OS) was defined as the time from the date of diagnosis to the date of death from any cause or the last follow-up date (July 31, 2025). Progression-free survival (PFS) was defined as the time from the start of treatment to the first recorded disease progression, recurrence, death, or the last follow-up date. Survival analyses were performed on the entire cohort, regardless of treatment received. Follow-up duration was calculated from the date of diagnosis to the last follow-up date (July 31, 2025) or death, and median follow-up time was 34.21 months.
7. Safety indicators: Incidence of grade ≥3 adverse events (CTCAE v5.0) during first-line treatment.

### 2.5 Statistical analysis

Data analysis was performed using SPSS 29.0 software (SPSS Inc., Chicago, IL, USA). Continuous variables, confirmed to follow a normal distribution by the Shapiro-Wilk test, were expressed as mean ± standard deviation and compared between groups using independent samples t-tests. Categorical variables were expressed as frequencies (percentages) and compared between groups using the χ² test. Survival curves were plotted using the Kaplan-Meier method, and the log-rank test was used to compare survival differences between groups. A multivariate Cox proportional hazards model was employed to adjust for potential confounders and analyze the independent impact of MDT involvement on survival. A multivariate logistic regression model was used to analyze the independent risk factors affecting treatment completion rates. A two-tailed P < 0.05 was considered statistically significant.

## 3 Results

### 3.1 Baseline characteristics of the patient

In **Table 1**, we present a comparison of baseline characteristics between the MDT group (n=115) and the non-MDT group (n=105). No significant differences were observed in age (P = 0.746), gender distribution (P = 0.724), BMI (P = 0.449), smoking history (P = 0.876), drinking history (P = 0.871), hypertension status (P = 0.871), diabetes mellitus prevalence (P = 0.883), pathological pattern (P = 0.773), TNM stage (P = 0.773), or ECOG PS score (P = 0.494). All variables showed no significant differences between the two groups, indicating that the baseline characteristics were well-balanced. Specifically, there was no significant difference in pathological pattern (P = 0.773) and TNM stage (P = 0.653). The frequency of EGFR mutation positivity (P = 0.424) and ALK rearrangement (P = 0.741) did not differ significantly between the MDT and non-MDT groups. These results indicate that the baseline characteristics were well balanced between the MDT and non-MDT groups (**Table 1**).

**Table 1:** Comparison of baseline characteristics between two groups.

Parameter	MDT group (n=115)	Non-MDT group (n=105)	t/χ^2^	P
Age (years)	66.43 ± 10.48	66.89 ± 10.64	0.324	0.746
Gender [n(%)]			0.125	0.724
-Male	83 (72.17%)	78 (74.29%)		
-Female	32 (27.83%)	27 (25.71%)		
BMI (kg/m2)	23.68 ± 3.15	24.02 ± 3.44	0.759	0.449
Smoking history [n(%)]			0.024	0.876
-Yes	68 (59.13%)	61 (58.10%)		
-No	47 (40.87%)	44 (41.90%)		
Drinking history [n(%)]			0.026	0.871
-Yes	34 (29.57%)	30 (28.57%)		
-No	81 (70.43%)	75 (71.43%)		
Hypertension [n(%)]			0.004	0.951
-Yes	64 (55.65%)	58 (55.24%)		
-No	51 (44.35%)	47 (44.76%)		
Diabetes mellitus [n(%)]			0.022	0.883
-Yes	15 (13.04%)	13 (12.38%)		
-No	100 (86.96%)	92 (87.62%)		
Pathological pattern [n(%)]			0.083	0.773
-NSCLC	98 (85.22%)	88 (83.81%)		
-SCLC	17 (14.78%)	17 (16.19%)		
TNM stage [n(%)]			0.202	0.653
-I-II	45 (39.13%)	38 (36.19%)		
-III-IV	70 (60.87%)	67 (63.81%)		
ECOG PS score [n(%)]			0.467	0.494
-0-1	92 (80.00%)	80 (76.19%)		
-≥2	23 (20.00%)	25 (23.81%)		
EGFR mutation positive [n(%)]	34 (29.57)	26 (24.76)	0.638	0.424
ALK rearrangement positive [n(%)]	9 (7.83)	7 (6.67)	0.109	0.741

BMI, body mass index; ECOG PS, Eastern Cooperative Oncology Group Performance Status; NSCLC, Non-Small Cell Lung Cancer; SCLC, Small-Cell Lung Cancer; TNM, Tumor Node Metastasis.

### 3.2 Distribution of initial treatment regimens

In **Table 2**, we compare the distribution of initial treatment regimens between the MDT group and the non-MDT group. There were no significant differences observed in the rates of surgery with perioperative therapy (P = 0.548), definitive chemoradiation (P = 0.961), or systemic therapy overall (P = 0.582). However, within the systemic therapy category, there were significant differences noted. Specifically, the use of targeted therapy was more frequent in the MDT group compared to the non-MDT group (P = 0.059), although this difference did not reach statistical significance at the conventional threshold. Immunotherapy (± chemotherapy) was significantly more common in the MDT group (χ²=3.944, P = 0.047). Conversely, platinum-based chemotherapy only was significantly less common in the MDT group (χ²=12.132, P<0.001). These findings suggest that MDT-recommended treatment plans were associated with a higher utilization of immunotherapy and targeted therapies, while reducing reliance on platinum-based chemotherapy alone (**Table 2**).

**Table 2:** Comparison of distribution of initial treatment regimens between two groups [n(%)].

Parameter	MDT group (n=115)	Non-MDT group (n=105)	χ^2^	P
Surgery with perioperative therapy	28 (24.35%)	22 (20.95%)	0.360	0.548
Definitive chemoradiation	20 (17.39%)	18 (17.15%)	0.002	0.961
Systemic therapy	67 (58.26%)	65 (61.90%)	0.304	0.582
-Targeted therapy	22 (32.84%)	12 (18.46%)	3.565	0.059
-Immunotherapy (± chemotherapy)	25 (37.31%)	14 (21.54%)	3.944	0.047
-Platinum-based chemotherapy only	20 (29.85%)	39 (60.00%)	12.132	<0.001

Surgery with perioperative therapy: neoadjuvant therapy, surgery, and/or adjuvant therapy.

### 3.3 Treatment completion and conversion situation

In **Table 3**, we compare the treatment completion and conversion situations between the MDT group and the non-MDT group. A significantly higher proportion of patients in the MDT group completed their initial treatment as planned compared to those in the non-MDT group (χ²=9.727, P = 0.002). The rate of treatment conversion was significantly lower in the MDT group (χ²=10.654, P = 0.001). Additionally, for those who did undergo treatment conversion, the time until conversion was significantly longer in the MDT group (t=13.000, P<0.001). These findings suggest that patients managed under an MDT model were more likely to adhere to their initial treatment plans and experience fewer changes in their treatment regimens. Moreover, when treatment conversions do occur in the MDT group, they tend to happen later in the course of treatment (**Table 3**).

**Table 3:** Comparison of treatment completion and conversion situation between two groups [n(%)].

Parameter	MDT group (n=115)	Non-MDT group (n=105)	t/χ^2^	P
Complete the initial treatment as planned	99 (86.09%)	72 (68.57%)	9.727	0.002
Treatment conversion [n(%)]	22 (19.13%)	41 (39.05%)	10.654	0.001
Treatment conversion time (months)	8.41 ± 2.19	5.16 ± 1.48	13.000	<0.001

### 3.4 Survival analysis results

**Figure 2:**
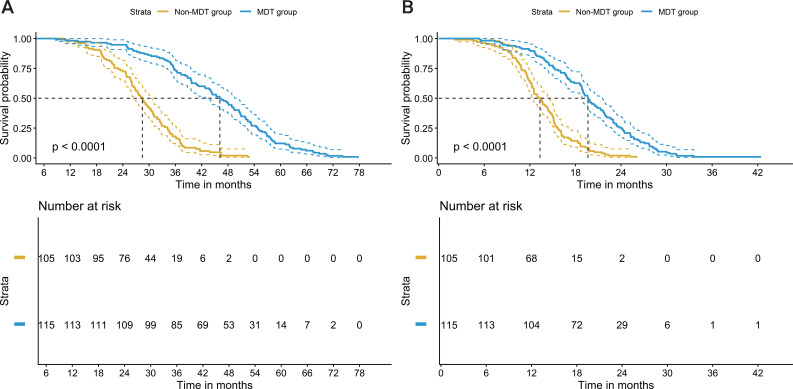
Comparison of survival time between two groups. **(A)** OS (months), Overall Survival; **(B)** PFS (months), Progression-Free Survival. Median OS: MDT 46.16 months (95% CI: 40.50–51.82), non-MDT 28.45 months (95% CI: 24.21–32.69); P<0.001. Median PFS: MDT 19.65 months (95% CI: 17.12–22.28), non-MDT 13.33 months (95% CI: 11.50–15.16); P<0.001.

In **Figure 2**, we present the comparison of survival times between the MDT group and the non-MDT group. The results indicate significant differences in both OS and PFS between the two groups. For OS, patients in the MDT group had a significantly longer survival time compared to those in the non-MDT group (t=10.891, P<0.001). Similarly, for PFS, the MDT group showed a significantly longer duration before disease progression (t=8.745, P<0.001). These findings highlight that adherence to MDT-recommended treatment plans was associated with significantly improved survival outcomes, both in terms of overall survival and progression-free survival (**Figure 2**).

### 3.5 Multivariate Cox regression analysis

In the multivariate Cox regression analysis examining factors affecting overall survival of lung cancer patients, several significant variables were identified. Participation in a MDT for treatment planning was associated with significantly improved survival (P = 0.009), with a HR of 0.605. Advanced TNM stage (III-IV vs I-II) and higher ECOG performance status score (≥2 vs 0-1) showed significant association with poorer survival outcomes, with HRs of 2.380 (P<0.001) and 1.923 (P = 0.002), respectively. Initial treatments involving targeted/immunotherapy or surgery/radiotherapy compared to platinum-based chemotherapy alone also demonstrated significant benefits on survival, showing HRs of 0.425 (P<0.001) and 0.302 (P<0.001), respectively. No significant differences were observed regarding age (P = 0.072) and pathological pattern (NSCLC vs SCLC, P = 0.068). The results underscore the potential benefits of integrating MDT recommendations into clinical practice for optimizing patient outcomes (**Table 4**).

**Table 4:** Multivariate Cox regression analysis affecting the overall survival of lung cancer patients.

Variables	β	Standard error	Wald χ²	P	HR (95% CI)
MDT participation (Yes vs No)	-0.502	0.192	6.823	0.009	0.605 (0.415-0.882)
Age (years)	0.018	0.010	3.240	0.072	1.018 (0.998-1.039)
Pathological pattern (NSCLC vs SCLC)	0.421	0.231	3.323	0.068	1.523 (0.968-2.395)
TNM stage (III-IV vs I-II)	0.867	0.205	17.876	<0.001	2.380 (1.593-3.556)
ECOG PS score (≥2 vs 0-1)	0.654	0.215	9.256	0.002	1.923 (1.262 - 2.931)
Initial treatment (Reference platinum-chemo only).					
-Targeted/Immunotherapy	-0.856	0.238	12.928	<0.001	0.425 (0.267 - 0.677)
-Surgery/Radiotherapy	-1.198	0.265	20.449	<0.001	0.302 (0.180 - 0.507)

HR, Hazard Ratio; CI, Confidence Interval; MDT, Multidisciplinary Team; NSCLC, Non-Small Cell Lung Cancer; TNM, Tumor Node Metastasis; ECOG PS, Eastern Cooperative Oncology Group Performance Status; SCLC, Small-Cell Lung Cancer.

### 3.6 Compliance analysis within the MDT group

**Figure 3:**
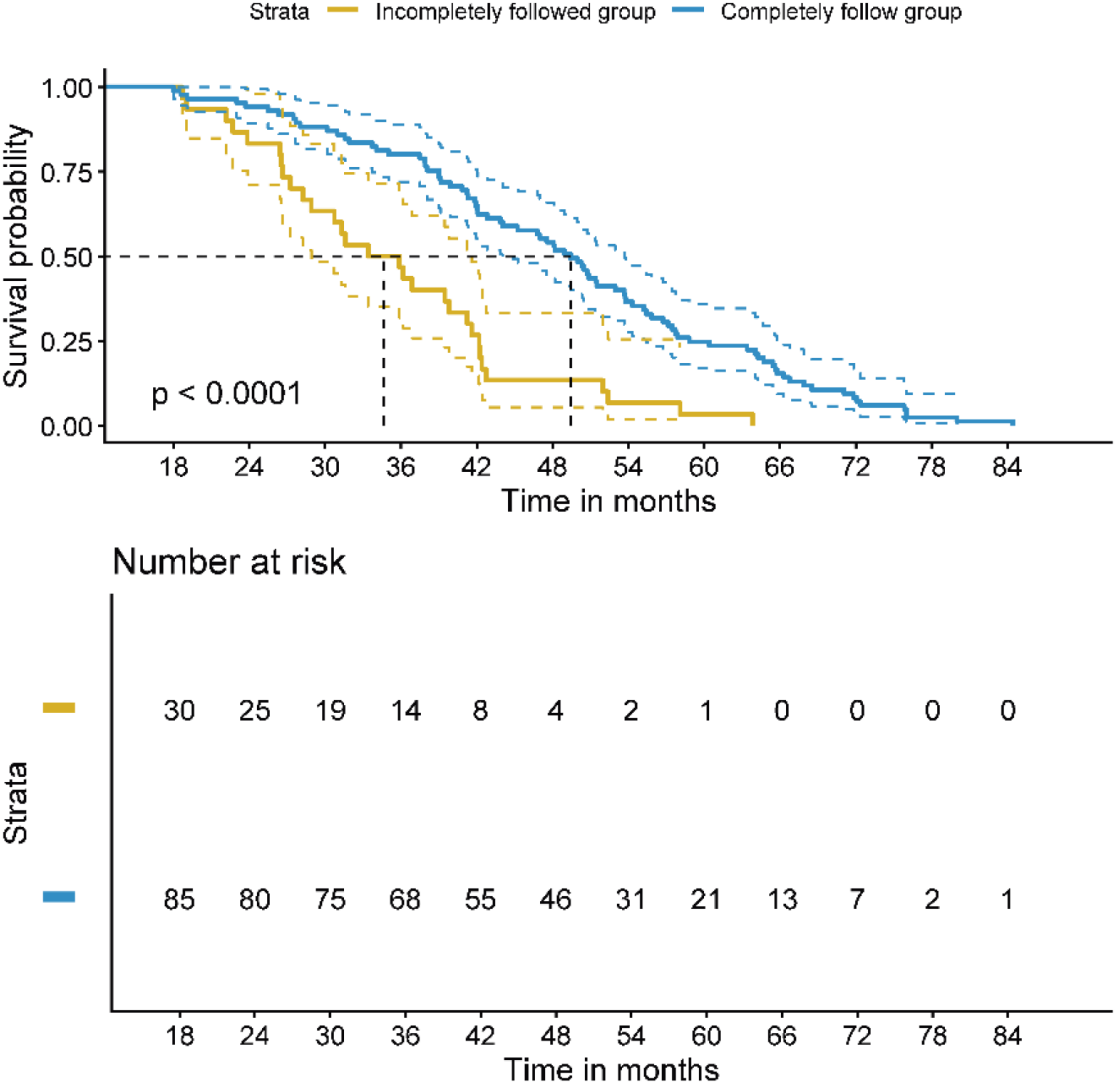
Survival comparison of patients with different compliance within the MDT group. OS, Overall Survival. Median OS: complete adherence group 49.42 months (95% CI: 43.20–55.64), incomplete adherence group 34.63 months (95% CI: 29.80–39.46); P<0.001.

In **Figure 3**, we present the survival comparison of patients with different levels of compliance within the MDT group. The analysis distinguishes between patients who completely followed MDT recommendations (n=85) and those who incompletely followed the recommendations (n=30). Patients who completely followed the MDT-recommended treatment plans had significantly longer OS compared to those who did not fully adhere to these recommendations (t=7.410, P<0.001). This indicates that full adherence to MDT treatment plans was associated with a substantial improvement in survival outcomes (**Figure 3**).

### 3.7 Treatment-related adverse events

In **Table 5**, we compare the incidence of treatment-related adverse events between the MDT group and the non-MDT group. No significant differences were observed in the rates of hematological toxicity (P = 0.387), gastrointestinal reactions (P = 0.395), liver and kidney function damage (P = 0.488), or any grade 3–4 event (P = 0.337). The comparable rates of adverse events suggest that the MDT approach did not increase the risk of adverse events, supporting the safety profile of MDT-directed care.

**Table 5:** Comparison of treatment-related adverse events between two groups [n(%)].

Parameter	MDT group (n=115)	Non-MDT group (n=105)	χ^2^	P
Hematological toxicity	28 (24.35%)	31 (29.52%)	0.749	0.387
Gastrointestinal reaction	15 (13.04%)	18 (17.14%)	0.723	0.395
Liver and kidney function damage	8 (6.96%)	10 (9.52%)	0.482	0.488
Any grade 3–4 event	42 (36.52%)	45 (42.86%)	0.921	0.337

### 3.8 Multivariate logistic regression analysis

In the multivariate logistic regression analysis of factors affecting the completion of treatment as planned (**Table 6**), participating in the MDT was associated with a significantly higher likelihood of completing treatment as planned (OR = 3.438, P = 0.002). Patients at advanced TNM stages (III-IV) had a lower likelihood of completing treatment compared to those at earlier stages (I-II) (OR = 0.410 P = 0.022). A worse ECOG PS score (≥2) was associated with a significantly lower likelihood of treatment completion (OR = 0.347, P = 0.017). Lastly, the initial use of targeted or immunotherapy treatments was found to increase the odds of completing treatment as planned (OR = 2.231, P = 0.009). Age did not show a significant association with the completion of treatment as planned (P = 0.674). The strong association between MDT participation and treatment completion suggested that collaborative approaches in patient care can enhance treatment adherence and potentially improve patient outcomes (**Table 6**).

**Table 6:** Multivariate logistic regression analysis of factors affecting the completion of treatment as planned.

Variables	β	Standard error	Wald χ²	P	OR (95% CI)
MDT participation (Yes vs No)	1.235	0.402	9.432	0.002	3.438 (1.563-7.562)
Age (years)	-0.008	0.019	0.177	0.674	0.992 (0.956-1.030)
TNM stage (III-IV vs I-II)	-0.892	0.388	5.285	0.022	0.410 (0.191-0.879)
ECOG PS score (≥2 vs 0-1)	-1.058	0.445	5.655	0.017	0.347 (0.145-0.831)
Initial treatment (Targeted/Immunotherapy vs others)	0.802	0.305	6.912	0.009	2.231 (1.234 - 4.052)

OR, Odds Ratio; CI, Confidence Interval; MDT: Multidisciplinary Team; TNM, Tumor Node Metastasis; ECOG PS, Eastern Cooperative Oncology Group Performance Status.

## 4 Discussion

The management of lung cancer, given its complexity and rapidly evolving treatment options, increasingly relies on structured multidisciplinary expertise through MDT models. While previous large cohort studies have demonstrated the survival benefit of MDT, our study provides additional insights into process-related outcomes such as treatment adherence and conversion. Our study adds to the growing body of evidence by demonstrating that treatment decisions formulated within an MDT framework are associated with several meaningful advantages throughout the patient care continuum, from initial strategy selection to long-term survival outcomes.

A primary observation from our study is the distinct pattern of initial treatment selection between the MDT and non-MDT groups. The MDT-recommended plans were associated with a higher utilization of contemporary systemic therapies, including immunotherapy and targeted agents, while the use of platinum-based chemotherapy alone was less frequent (14, 15). This shift aligns with the core objective of MDT discussions: to synthesize comprehensive diagnostic information—including pathology, molecular profiling, and imaging—into a personalized, stage-appropriate, and evidence-based plan (17, 18). Collaborative review minimizes the risk of oversight, such as missing an indication for biomarker testing or a potential surgical option (19, 20). For instance, research has shown that MDT discussion for stage III non-small cell lung cancer (NSCLC) can result in a substantial survival benefit. This benefit likely stems, in part, from the MDT’s role in ensuring patients receive the most current and potentially effective therapies available, rather than defaulting to a more familiar but less personalized approach (21).

Beyond initial planning, the MDT process appeared to enhance treatment pathway stability. Our findings indicate that patients in the MDT group were more likely to complete their initial treatment plan as scheduled and experienced fewer unplanned treatment conversions. When conversions did occur, they happened later in the treatment course. This greater adherence can be attributed to several factors inherent to the MDT model. First, a plan forged through consensus among surgeons, medical oncologists, and radiation oncologists may carry greater weight and clarity for the treating physician and the patient, reducing ambiguity (22). Second, the MDT process often includes discussing and planning for potential future steps (e.g., next-line therapy upon progression), creating a more coherent long-term strategy (12). Furthermore, studies have linked MDT management to shorter waiting times for key diagnostic and treatment procedures. This improved logistical efficiency reduces delays that can lead to clinical deterioration or patient anxiety, thereby supporting the uninterrupted execution of the planned therapy (23, 24).

The association between MDT-guided care and improved overall and progression-free survival was the most significant finding, corroborated by other studies (25). This survival advantage is unlikely to be the result of a single factor but rather the culmination of the aforementioned effects: more precise initial treatment selection, higher adherence to optimal therapy, and better-managed care transitions. The MDT creates an ecosystem where complex cases benefit from collective decision-making, which is particularly crucial for stages with multiple valid treatment options, such as stage III disease (26). Importantly, our multivariate analysis positioned MDT participation as an independent factor associated with favorable survival, even after accounting for stage, performance status, and treatment type. The higher use of modern systemic therapies in the MDT group may reflect treatment selection bias, as patients with certain molecular profiles or PD-L1 expression levels may have been preferentially referred for MDT discussion. However, multivariate Cox regression adjusted for treatment type, and MDT participation remained an independent predictor of survival. This suggests that the survival benefit associated with MDT is not solely attributable to the specific therapies administered, but rather reflects a broader impact of coordinated, multidisciplinary care (27).

An internal analysis within our MDT group provided further insight: patients who fully adhered to the MDT-recommended plan had more favorable survival outcomes compared to those whose actual treatment deviated from it (28). This observation underscores that the survival benefit is intrinsically linked to the implementation of the collaborative decision. Deviations from consensus recommendations may occur due to patient preference, unforeseen toxicity, or evolving clinical status. However, this finding highlights the importance of not only conducting the MDT meeting but also ensuring effective communication of the rationale to the patient and facilitating access to the recommended therapies (7). It emphasizes that the MDT’s impact is realized at the point of care delivery (15).

Our analysis found that the rates of treatment-related adverse events were comparable between the MDT and non-MDT groups. This is a critical point, as it indicates that the more frequent use of novel therapies like immunotherapy within the MDT framework did not come at the cost of increased severe toxicity. This safety profile supports the feasibility of implementing MDT-driven treatment intensification or novel combinations in real-world settings (29, 30). However, it is essential to acknowledge the challenges and resource intensities associated with running effective MDTs. Literature points to issues such as time constraints, rising caseloads, the need for strong coordination, and the potential for variable implementation quality across different centers. For example, one study evaluating the consistency of MDT decisions found only moderate agreement on tumor staging and poor agreement on treatment intent for complex stage III cases across different centers. These factors can limit the generalizability and full potential of the MDT model (31).

This study has several limitations that should be acknowledged. As a single-center, retrospective analysis, it may be subject to selection bias and its findings may not be fully generalizable to other healthcare settings with different resource allocations or MDT structures. Due to the retrospective design, we lacked complete data on molecular profiles, detailed tumor burden, and comorbidity indices. However, baseline characteristics were well balanced between groups, and multivariate analyses adjusted for key confounders. The assignment to MDT discussion was not randomized, and while baseline characteristics were balanced, unmeasured confounding factors may exist. Additionally, in the non-MDT group, some patients received urgent surgery or radical radiotherapy without prior MDT discussion due to clinical urgency or direct referral. While this reflects real-world practice, such patients may have distinct prognostic profiles, which could introduce confounding in survival comparisons. We did not perform stage-stratified analyses due to limited sample size, which could be explored in larger prospective studies. The longer time from diagnosis to treatment in the MDT group may introduce immortal time bias. However, survival analysis from the start of treatment partially mitigates this concern. Furthermore, our study did not capture detailed data on the reasons for non-adherence to MDT recommendations or the specific logistical pathways that led to improved treatment completion. Future research should focus on prospective, multi-center designs to validate these findings. It is crucial to move beyond demonstrating that MDTs are beneficial and investigate how to optimize them. Studies should explore standardized protocols for MDT conduct, the integration of molecular tumor boards for complex genomic data interpretation, and the use of digital tools to improve efficiency and data handling. Additionally, incorporating patient-reported outcomes and cost-effectiveness analyses into the evaluation of MDT models will provide a more comprehensive understanding of their value. Finally, as treatment options continue to expand—with novel combinations like antibody-drug conjugates plus immunotherapy showing promise—the role of the MDT in navigating these increasingly complex landscapes will only become more vital.

## 5 Conclusion

In conclusion, our study provides evidence that a formal MDT model in lung cancer care influences the treatment pathway positively at multiple stages, leading to more precise initial therapy, better adherence, and ultimately, improved survival outcomes. These benefits appear to stem from the synergistic effect of collaborative decision-making and coordinated care execution. Despite existing challenges, the MDT remains a cornerstone of high-quality, personalized oncology care.

## Data Availability

The raw data supporting the conclusions of this article will be made available by the authors, without undue reservation.
